# Changes in T-lymphocyte subpopulations in patients with colorectal cancer before and after acupoint catgut embedding acupuncture observation

**DOI:** 10.1515/biol-2022-1060

**Published:** 2025-05-20

**Authors:** Caide Yang, Jinlian Bao, Dengke Li

**Affiliations:** Department of Integrated Traditional Chinese and Western Medicine, Donggang Hospital, The First Hospital of Lanzhou University, Lanzhou, 730020, China; Department of Pharmacy, Donggang Hospital, The First Hospital of Lanzhou University, Lanzhou, 730020, China

**Keywords:** colorectal cancer, acupoint catgut embedding, T lymphocytes, immunoregulation

## Abstract

This study aims to explore changes in peripheral blood T-lymphocyte subsets in colorectal cancer (CRC) patients before and after acupoint catgut embedding (ACE) acupuncture observation. Eighty CRC surgical patients who visited from April 2017 to May 2022 were selected as study samples using the convenience sampling method. Patients were randomly divided into a control group (*n* = 40, receiving conventional chemotherapy) and an observation group (*n* = 40, receiving 4 weeks of additional ACE acupuncture). The observation group showed higher rates of complete and partial remission compared to the control, though the difference was not statistically significant (*P* > 0.05). The observation group experienced less reduction in CD^3+^, CD^4+^ T lymphocytes, and natural killer cells during chemotherapy. Compared with the control group, the percentage of CD^3+^ and CD^4+^ T cells in the observation group significantly increased after the intervention, while CD^8+^ T-cell levels decreased. The CD^4+^/CD^8+^ ratio was at a higher level, and the discrepancy was statistically obvious (*P* < 0.05). Acupuncture therapy can maintain the normal distribution of peripheral blood T-lymphocyte subpopulations during chemotherapy in patients with CRC, thus better maintaining the immune status of patients.

## Graphical abstract

**Figure:**
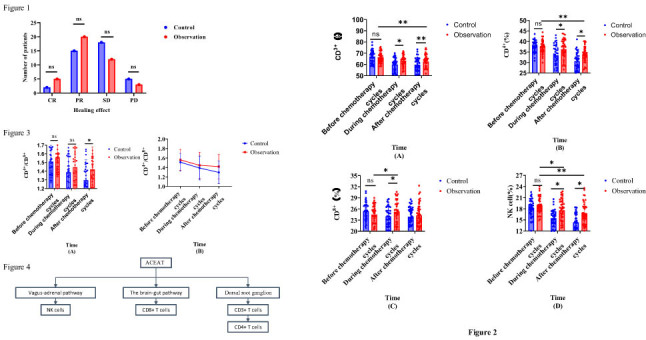

## 1 Introduction

Colorectal cancer (CRC) is one of the malignant tumors worldwide, with a high incidence rate and high recurrence rate [1]. CRC patients often experience postoperative recurrence due to residual small tumors and host cell immune dysfunction, which seriously affects their clinical efficacy and quality of life [2]. Postoperative neoadjuvant chemotherapy (PNC) is a commonly used observation method in clinical practice to prevent CRC recurrence, which can reduce patient mortality [3]. Oxaliplatin, fluorouracil, and capecitabine [4] are commonly used drugs for PNC [5]. As an increasingly common observation option for CRC patients, PNC faces challenges such as limited tumor targeting and common side effects such as gastrointestinal reactions and can also cause immune dysfunction in patients [6].

Currently, oxaliplatin is among the most effective chemotherapy drugs used for CRC observation [7]. During the postoperative chemotherapy stage, adjuvant immunotherapy is usually used for immune regulation, such as 5-fluorouracil or low-dose cyclophosphamide, to strengthen the immune function of the body by inhibiting immunosuppressive cells [8]. The anti-tumor immune response depends on the interaction between tumor cells and immune cells [9]. CRC patients can benefit from immunotherapy strategies that stimulate effector T-cell action [10]. Chemotherapy can lead to a decrease in CD^4+^ T lymphocytes and a rise in infiltration of CD^8+^ T lymphocytes [11]. CD^8+^ T lymphocytes have chemotaxis toward CRC [12]. Intraepithelial lymphocytes play a crucial role in maintaining gastrointestinal epithelial cells and are important cells for the defense of intestinal tumors [13]. Therefore, PNC is an important means to prevent postoperative recurrence of CRC, but it is still necessary to adjust immune function during chemotherapy to reduce its observation side effects.

In the past decade, clinical research on acupoint catgut embedding (ACE) has developed rapidly and has gradually formed relatively standardized and unified operating standards [14]. Moreover, ACE can improve the immune activity of respiratory system cells and reduce lung fiber content [15]. Animal experimental results show that acupoint catgut embedding therapy (ACET) can improve various inflammatory factors in the local microenvironment of rats [16] and has been used in the immune regulation process of liver cancer mice [17]. However, the study on the application of CRC patients is limited, and it is widely used in ulcerative colitis with precancerous lesions [18]. Research has found that acupuncture therapy can bi-directional regulate peripheral blood lymphocytes and neutrophils, thereby improving T-lymphocyte subsets (T-lps) and natural killer (NK) cells, thereby promoting the recovery of immune function in patients after CRC surgery [19]. Acupoint catgut embedding acupuncture therapy (ACEAT) is a new observation technique that combines the advantages of ACET and acupuncture therapy [20]. Based on previous research results, it can be concluded that ACE combined with acupuncture observation can regulate the proportion of T-lps, which is of great significance for improving the body’s immunity.

Cytokines are involved in the process of tumour progression, especially in the context of disturbances in the human tumour microenvironment, where a series of changes in cytokines occur [21]. NK cells are highly regarded for their potential therapeutic value in cancer [22]. At the same time, cytokines are essential for the maturation, activation, and survival of NK cells [23]. To further improve the T-lps ratio in postoperative CRC patients, this study aims to conduct ACEAT for 4 weeks and observe the changes in peripheral blood T-lps ratio before and after observation. Based on the current situation that peripheral blood T-lps imbalance can lead to weakened anti-tumor immune response and poor prognosis in CRC, the research group hypothesizes that ACEAT can adjust the immune status of CRC cases. The specific manifestation is to increase the CD^3+^/CD^4+^ T lymphocytes, reduce the CD^8+^ T, and make the CD^4+^/CD^8+^ ratio more balanced. This study can provide a new theoretical basis for the utility of ACEAT in tumor immunity.

## 2 Materials and methods

### 2.1 General data

The convenience sampling method selected 80 CRC patients who visited from April 2017 to May 2022 as the study samples. The patients were divided into a control group and an observation group using the red and blue ball method, with 40 patients in each group. Inclusive criteria are as follows: (1) patients diagnosed with colorectal adenocarcinoma through postoperative pathology and treated with colorectal tumor resection surgery; (2) without radiotherapy and chemotherapy observation; (3) Karnofsky physical condition score ≥70 points; (4) expected survival time ≥6 months; and (5) sign an informed consent form. Exclusive criteria are as follows: (1) complication with severe heart, lung, liver, kidney, and other important organ diseases; (2) accompanied by severe coagulation dysfunction or autoimmune diseases; (3) concomitant severe mental illness; (4) recently, received acupuncture and moxibustion observation; (5) poor compliance and inability to complete the experiment; and (6) allergies to the drugs required for the experiment. There was *P* > 0.05 in gender, age, BMI, tumor stage, tumor site, and distribution of main symptoms among patients, indicating a baseline data balance (Table 1).

**Table 1:** Comparative data of general characteristics among patients

Group (*n*)	Gender (*n*)		Age (years, \[\bar{x}]\] ± *s*)	BMI (kg/m^2^, \[\bar{x}]\] ± *s*)	Tumor staging (*n*)		Main symptoms (*n*)			Tumor site (*n*)	
	Male	Female			IIB	IIIC	Anemia	Diarrhea	Constipation	Colon	Rectum
Control (40)	25	15	56.1 ± 8.4	24.1 ± 2.4	25	15	13	15	12	18	22
Observation (40)	24	16	55.8 ± 7.5	23.1 ± 2.8	27	13	10	18	12	19	21
*χ* ^*2*^/*t*	0.053		0.126	0.039	0.220		0.664			1.251	
*P*	0.818		0.900	0.969	0.639		0.717			0.263	


**Informed consent:** Informed consent has been obtained from all individuals included in this study.
**Ethical approval:** The research related to human use has been complied with all the relevant national regulations and, institutional policies and in accordance with the tenets of the Helsinki Declaration, and has been approved by the Medical Ethics Committee of our institution (Donggang Hospital, The First Hospital of Lanzhou University, No. LZ02016DG12).

### 2.2 Research method

#### 2.2.1 Control group

Routine observation: on the first day of chemotherapy, 130 mg/m^2^ oxaliplatin (produced by Sanofi Syntherabo France) was administered intravenously, with an intravenous infusion time controlled within 2 h. On the 1st to 14th days of chemotherapy, 1,000 mg/m^2^ capecitabine tablets (produced by Shanghai Roche Pharmaceutical Co., Ltd.), bid, were administered orally. Each chemotherapy cycle in this study lasted 21 days. After the end of one cycle, rest for 7 days and start the next chemotherapy cycle. Chemotherapy is given in conjunction with antiemetic, liver protective, anemia correction, and white blood cell elevation observation and provide nutritional support observation if necessary. When the patient experiences serious adverse reactions or disease progression, chemotherapy should be terminated or other drugs replaced for chemotherapy. At the same time, fake acupuncture and moxibustion intervention are carried out, that is, acupuncture was conducted at 0.5–5 cm beside the “Left Outer Mausoleum, Left Sliding Meat Gate, Guanyuan, Qihai, Zhongwan, Xiawan, Right Outer Mausoleum, and Right Sliding Meat Gate” points, or between the two meridians. After inserting the needle will insert but not be used to avoid getting air. The other methods are similar to the observation group.

#### 2.2.2 Observation group

Perform 4-week ACEAT on the basis of the control group. First, select Left Outer Mausoleum, Left Sliding Meat Gate, Guanyuan, Qihai, Zhongwan, Xiawan, Right Outer Mausoleum, and Right Sliding Meat Gate as ACE acupoints. For routine disinfection of the patient’s skin, use sterile scissors to cut the medical catgut into a length of approximately 1 cm for future use. A No. 8 disposable syringe needle serves as the sleeve, with a No. 28 stainless steel needle used as the core. A section of the thread body is placed on the front end of the buried needle using tweezers. Tweezers serve as a pushing tool to push the segment of the sheep intestine into the needle tube sleeve. Use a filiform needle to pierce the acupoint, and after obtaining qi, push the needle core while exiting the needle tube, so that the sheep intestines can be accurately buried in the acupoint. Perform once before chemotherapy for 7–10 days. Then, remove the catgut under sterile operation. Afterwards, acupuncture observation is carried out. The main acupoints are Shenguan, Taichong, Taixi, Yanglingquan, Shangjuxu, Neiguan, Sanyinjiao, and Zusanli. Use 0.30 mm × 40 mm millineedles. Among them, the three acupoints of Zusanli, Taixi, and Shenguan are injected with the twisting and lifting interpolation method, while the other acupoints use the leveling and reducing method. Inject once every 10 min and keep the needle for 20 min. Once a day, take 2 days off per week for a total of 3 weeks, and the total course of ACEAT is 4 weeks.

### 2.3 Observed indicators

#### 2.3.1 General information

A general data collection table is used to record the patient’s age, gender, lesion location, BMI, tumor stage, and main symptoms. The data information was collected from the patient’s medical records and was extracted and recorded by two researchers. In cases of data inconsistency, a third researcher reviews and corrects the data.

#### 2.3.2 Clinical effect

Evaluate the postoperative tumor reduction effect on patients and evaluate the clinical efficacy of both groups at 4 weeks after surgery. The therapeutic effect is divided into complete remission (CR): the tumor completely disappears and lasts for more than 4 weeks. Partial remission (PR): the maximum diameter of the tumor decreases by more than 30% and lasts for more than 4 weeks. Disease stability (SD): reduce insufficient PR and increase no more than PD. Disease progression (PD): the maxtumor diameter rises by more than 20% or new lesions appear.

#### 2.3.3 T lymphocytes

Sample collection: 2 ml of blood is drawn from the elbow vein on an empty stomach in the morning and EDTA is added as an anticoagulant during the before, during, and after chemotherapy cycles. The two were thoroughly mixed. This study uses a BD FACSCALIBUR flow cytometer equipped with CellQuest software for data analysis. Analysis method: 100 μl of whole blood sample is taken, corresponding monoclonal antibodies (mAbs) are added, and incubated for 15 min. 10% fetal bovine serum is added to the complete culture medium to ensure a final volume of 100 μl. Red blood cell lysis and washing are performed and resuspended in phosphate-buffered saline (PBS) for machine detection. The determination of CD^3+^, CD^4+^, and CD^8+^ T cells is performed using a combination of mAbs. The determination of NK cells is carried out using a combination of CD3FITC/CD16CD56PE mAbs. 20 μl of two appropriate combinations of mAbs is added to each sample tube and incubated at 4°C for 30 min to allow the mAbs to bind to the corresponding antigens on the cell surface. Cells are washed twice with cold PBS to remove unbound antibodies, and red blood cell lysis is performed to eliminate interference from red blood cells. Cells are fixed with 1% formaldehyde to maintain cell structure and reduce degradation. The loss cell analyzer is applied for analysis, the detector voltage is set according to the same type of control tube, and compensation is adjusted using a single positive tube to reduce spectral overlap or fluorescence leakage. A total of 10,000 gated events are collected. These events are validated as PBMCs based on physical characteristics (FSC and SSC) and CD45 expression (>95% CD^45+^ cells), and data analysis is performed using CellQUEST software. Based on the gating strategies of CD45 and SSC/FSC, the T-cell population is determined and further subdivided into CD^4+^ and CD^8+^ T-cell subsets. NK cells are analyzed based on the expression of CD3 and CD16/CD56. Detection indicators: Detect the percentage of CD^3+^, CD^4+^, and CD^8+^ T lymphocytes. Quality control: Set the same type of control sample and adjust the four quadrants of the volume scatter plot to ensure accurate clustering. Read the results and record the percentage of T lymphocytes in different subgroups. Calculate the CD^4+^/CD^8+^ ratio.

### 2.4 Statistical methods

All data were analyzed using SPSS 22.0. The measurement data were represented by \[\bar{x}]\] ± *s*, and independent sample *t*-tests were utilized for comparison between the two groups. Paired *t*-tests were taken to compare between the two time points. The comparison of counting data adopted a *χ* ^2^-test. Draw a bar chart using GraphPad software to display the ratio of T lymphocytes in different subpopulations. All tests were bilateral tests. *P* < 0.05 means statistically significant differences. Pearson correlation analysis was used to determine the correlation between ACEAT treatment and T-lymphocyte subsets and NK cells.

## 3 Results

### 3.1 Clinical efficacy of patients

**Figure 1:**
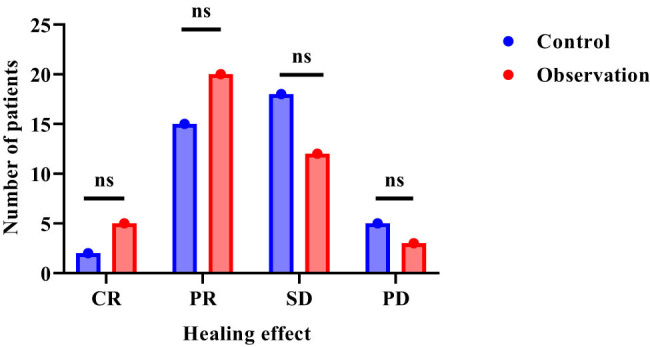
Comparative results of clinical efficacy. Note: “ns” is *P* > 0.05.

In Table 2, compared with the control, the observation group had more cases of CR and PR and fewer cases of SD and PD. Although the observation group had better efficacy evaluation indicators than the control group, the differences in CR, PR, SD, and PD between the groups did not reach statistical significance. In Figure 1, the therapeutic effect of patients in the observation group is concentrated on PR, while the control group is more concentrated on SD. The observation group has an obvious improvement.

**Table 2:** Comparison of clinical efficacy (*n* [%])

Group (*n*)	CR	PR	SD	PD
Control (40)	2 (5.0)	15 (37.5)	18 (45.0)	5 (12.5)
Observation(40)	5 (12.5)	20 (50.0)	12 (30.0)	3 (7.5)
*Z*	−1.871			
*P*	0.061			

### 3.2 Changes in T lymphocytes in patients

**Figure 2:**
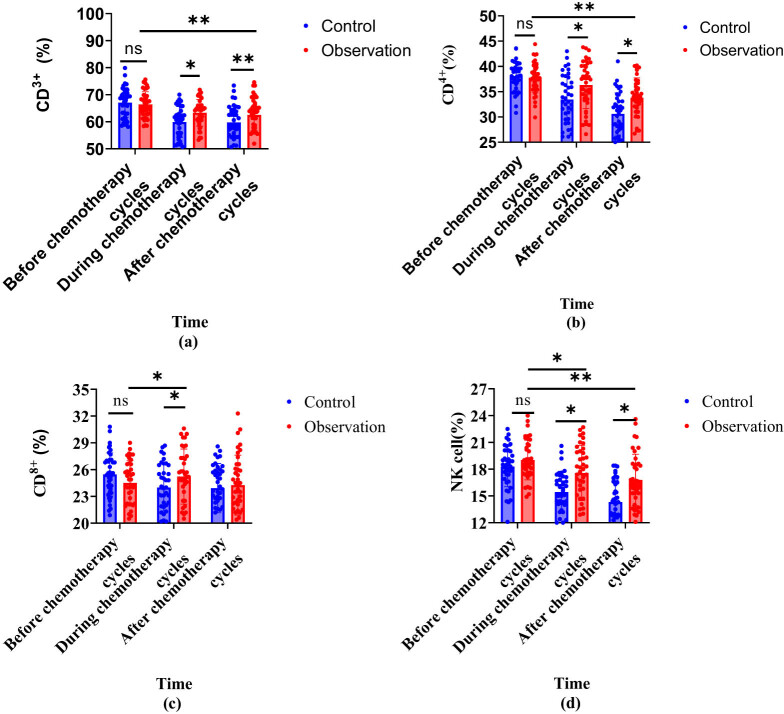
Trend of T-lymphocyte changes in two groups of patients. Note: (a) CD^3+^ (%); (b) CD^4+^ (%); (c) CD^8+^ (%); and (d) NK cells (%).

Compared with the control, the observation group had a higher percentage of CD^3+^, CD^8+^ T lymphocytes, and NK cells on the 7th day of chemotherapy, and a higher percentage of CD^4+^ T lymphocytes on the 4th week of chemotherapy, with a statistically obvious difference (*P* < 0.05). Compared with before and after chemotherapy, the observation group showed a decrease in CD^3+^, CD^4+^ T cells and NK cells on the 7th and 4th weeks of chemotherapy compared to before, while CD^8+^ T lymphocytes increased on the 7th day of chemotherapy compared to before, with a statistically significant difference (*P* < 0.05). This proved that in contrast with the control, the observation showed a lighter degree of inhibition of T-lps during chemotherapy (Table 3). In Figure 2, the observation group showed a slow downward trend in CD^3+^ (%), CD^4+^ (%), and NK cells (%), with a slower decline compared to the control group and CD^8+^ (%) tended to first increasing and then decreasing.

**Table 3:** Comparison of T-lymphocyte changes [ \[\bar{x}]\] ± *s*]

Group	Time	CD^3+^ (%)	CD^4+^ (%)	CD^8+^ (%)	NK cells (%)
Control	Before chemotherapy cycles	67.1 ± 5.5	38.1 ± 2.8	25.5 ± 2.4	18.3 ± 2.3
	During chemotherapy cycles	59.9 ± 5.7	33.5 ± 4.8	24.4 ± 3.3	15.4 ± 2.3
	After chemotherapy cycles	59.8 ± 6.1	30.6 ± 4.2	23.9 ± 2.8	14.3 ± 2.5
Observation	Before chemotherapy cycles	66.4 ± 4.7	37.8 ± 3.0	24.5 ± 2.4	19.0 ± 2.2
	During chemotherapy cycles	63.3 ± 4.9*^#^	36.3 ± 4.7*	25.6 ± 3.5*^#^	17.6 ± 3.1*^#^
	After chemotherapy cycles	62.5 ± 7.0^#^	33.8 ± 3.9*^#^	24.3 ± 3.4	16.8 ± 2.9*^#^

Note: Compared with the control during the same period, *P < 0.05. Compared with before chemotherapy in this group, ^#^ *P* < 0.05.

### 3.3 Changes in CD^4+^/CD^8+^ ratios between two groups

**Figure 3:**
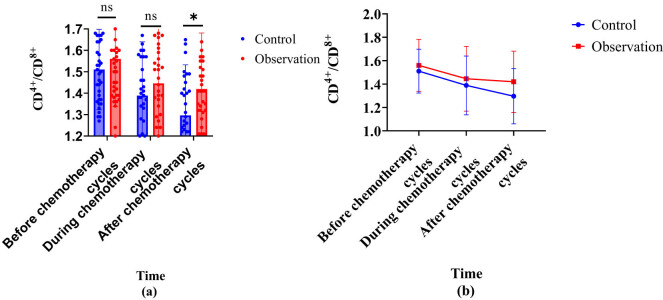
Changes in CD^4+^/CD^8+^ in two groups of patients. Note: (a) is a comparison bar chart between CD^4+^/CD^8+^ groups and (b) line chart comparing CD^4+^/CD^8+^ groups.

In Figure 3, there was no statistically significant discrepancy in the CD^4+^/CD^8+^ ratio between the two groups before chemotherapy. On the 7th day of chemotherapy, the CD^4+^/CD^8+^ ratio in both groups decreased compared to before chemotherapy, but there was *P* > 0.05 between the groups. In the 4th week of chemotherapy, the CD^4+^/CD^8+^ ratio in the control group significantly decreased compared to before and on the 7th day of chemotherapy, while there was no statistically significant difference between the observation group and before chemotherapy. The comparison of CD^4+^/CD^8+^ ratio at week 4 of chemotherapy showed that the observation group greatly outperformed the control. The results indicate that the observation group can inhibit the decrease in the CD^4+^/CD^8+^ ratio caused by chemotherapy.

### 3.4 The correlation between ACEAT, T lymphocytes, and NK cells

Before chemotherapy cycles, there was no significant correlation between T-lymphocyte subsets, NK cells, and ACEAT; during chemotherapy cycles and after chemotherapy cycles, there was a positive correlation (*P* < 0.01) between CD^3+^ (%), CD^4+^ (%), NK cells (%), and ACEAT. The detailed information is shown in Table 4.

**Table 4:** Correlation between ACEAT and T-lymphocyte subsets and NK cells at different time points

Time	CD^3+^ (%)	CD^4+^ (%)	CD^8+^ (%)	NK cells (%)	CD^4+^/CD^8+^
Before chemotherapy cycles	−0.066	−0.056	−0.191	0.163	0.121
During chemotherapy cycles	0.307**	0.291**	0.172	0.367**	0.109
After chemotherapy cycles	0.206	0.372**	0.055	0.424**	0.243

Note: **P < 0.01.

### 3.5 The immune regulatory pathway of ACEAT on T lymphocytes and NK cells

**Figure 4:**
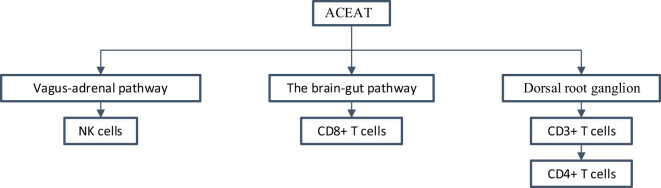
Immune regulatory pathways of ACEAT on T lymphocytes and NK cells.

Combined with the acupuncture and moxibustion system immune regulation mechanism diagram drawn by Wang et al. [24] and the research results in this article, the action path of ACEAT on T lymphocytes and NK cells is sorted out, as shown in Figure 4.

## 4 Discussion

This study observed the changes in peripheral blood T-lps in CRC patients undergoing routine chemotherapy combined with ACEAT. The results showed that patients who received additional ACEAT had less inhibition of CD^3+^, CD^4+^ T and NK cells during chemotherapy compared to the control group who only received conventional chemotherapy. This may be related to the immune regulatory effects of ACE and acupuncture observation. Research has shown that acupoint acupuncture stimulation can improve the Th1 cells, reduce the Th2 cells, and promote cellular immune balance in patients with advanced non-small-cell lung cancer (NSCLC) [25]. Therefore, ACET can alleviate the immuno-suppressive effect caused by chemotherapy. In addition, this study found that ACEAT can increase the ratio of CD^3+^ and CD^4+^ T cells after chemotherapy, reduce the CD^8+^ T cells, and maintain the CD^4+^/CD^8+^ ratio at a higher level. This indicates that ACE can regulate the normal distribution of T-lps. The reason for this is that a significant decrease in CD^4+^ T cells after chemotherapy is a manifestation of impaired immune function [26]. ACEAT can promote a balanced ratio of CD^4+^ and CD^8+^ cells, helping to maintain normal cellular immune function.

This study also found that receiving ACE observation during chemotherapy can alleviate the immune function decline caused by chemotherapy in CRC patients. Sun et al. [27] and Sui et al. [28] suggested that acupoint therapy can exert anti-tumor effects by regulating the intestinal microbiota, while this study only observed from an immunological perspective. In the future, a more comprehensive evaluation can be conducted by combining the detection of changes in gut microbiota. For example, Inprasit et al. [29] found through mouse model research that ACEAT can increase weight control. Wu et al. [30] found through meta-analysis that ACEAT can reduce abdominal obesity. Moreover, obesity is closely related to intestinal microbiota imbalance. Zhong et al. [31] and Zhang et al. [32] found through meta-analysis that ACE can effectively treat allergic rhinitis and insomnia, and can improve the immune function of insomnia patients. Both of these are immune-related diseases, supporting the immune regulatory effect of ACE, which is consistent with the conclusions of this study. In addition, Li et al. [33] and Gao et al. [34] used percutaneous electrical stimulation of acupoints such as Changshu and Zusanli in tumor patients to observe postoperative functional recovery-related indicators and also achieved positive results. This study adds ACE observation on the basis of acupuncture observation, with different intervention methods. Moreover, this study only focuses on changes in T lymphocytes, but the above literature suggests that more focus should be paid to the recovery of symptoms in the body. Overall, existing studies have shown that ACEAT can potentially enhance the immune status of tumor patients by regulating T lymphocytes, optimizing their sub-population distribution and function. However, further research is needed to confirm the specific mechanism.

Acupuncture and moxibustion may regulate the number of T-cell subsets and NK cells in the peripheral blood by activating the autonomic nervous system and regulating the immune system. Specifically, acupuncture and moxibustion stimulation of acupoints can act on the activities of the dorsal root ganglion under the skin, thus promoting the generation and activation of immune cells. In addition, acupuncture and moxibustion can regulate the expression of cytokines, such as increasing the levels of interleukin-4 and interferon-γ, which play an important role in the proliferation and functional regulation of immune cells. Through these mechanisms, acupuncture and moxibustion can promote the increase of the number of leukocyte subsets in the peripheral blood, including T cells, B cells, and NK cells.

T lymphocytes are essential in anti-tumor immune response. This study preliminarily confirms that ACEAT can alleviate the damage of chemotherapy to the immune function of CRC patients and help maintain the immune status of T lymphocytes. CD^4+^ T lymphocytes can differentiate into subgroups such as Th17 and Treg, participating in the immune response regulation of tumor microenvironment [35]. During the progression of the tumor or chemotherapy is a decrease in the percentage of cells [36]. This study observed an increase in the proportion of CD^4+^ T lymphocytes, suggesting that ACE may inhibit tumor growth by promoting Th17 differentiation. Inhibiting CD^4+^ T-lymphocyte activation and proliferation can further inhibit anti-tumor immune response [37]. P53 can negatively regulate Sirt1 and promote tumor cell apoptosis, while Sirt1 can in turn reduce p53 activity through deacetylation [38]. This is because ACEAT may inhibit tumor growth by down-regulating the p53 pathway. The proportion of CD^3+^ and CD^8+^ T lymphocytes is related to tumor prognosis and immunotherapy responsiveness [39], and maintaining their normal distribution is beneficial for improving clinical efficacy. Previous studies have shown that acupuncture therapy can regulate the immune function of the body. For example, research has found that acupuncture combined with moxibustion can regulate the humoral and cellular immunity of patients with thoracic butterfly type high safety arteritis [40]. Other studies show that acupuncture and moxibustion can alleviate allergic airway inflammation by regulating the balance of CD^4+^ T-lymphocyte subtypes in experimental asthma mice [41]. At the same time, it was found that early application of fire needles combined with cupping therapy can treat acute herpes zoster by reducing Th17/Treg levels [42]. Acupuncture and moxibustion can improve the cognitive dysfunction of VA rats and regulate their peripheral immune function and inflammation [43]. It has been found that moxibustion can control asthma symptoms by regulating the imbalance of Th17/Treg cells in asthma patients [44]. Moxibustion can increase the amount of T-lps in peripheral blood [45]. Therefore, ACE, as an acupuncture therapy, its mechanism of regulating immune balance and improving T-lymphocyte population is similar to other acupuncture and moxibustion therapies. The results further confirm that ACE can increase the proportion of T-lps in the peripheral blood of colon cancer patients, especially CD^3+^ and CD^4+^ T cells, regulate the balance of Th1/Th2 cells, and thus exert anti-tumor effects. There is a positive correlation (*P* < 0.01) between CD^3+^ (%), CD^4+^ (%), NK cells (%) and ACEAT during and after chemotherapy cycles. This further confirms the positive regulatory effect of ACEAT on CD^3+^ (%), CD^4+^ (%), and NK cells (%) in this study. This article provides new ideas for the comprehensive observation of CRC and supports the application prospects of chemotherapy combined with ACE observation. In addition, this study revealed some mechanisms by which ACE regulates the immune function of tumor patients, laying the foundation for subsequent research.

### 4.1 Limitations of the study

However, the study’s sample size is small, and the statistical validity is limited. The conclusion still needs to be verified by expanding the sample size. The observation indicators are relatively single, only focusing on changes in T lymphocytes and not involving comprehensive immunological indicators. The mechanism research is lacking, and the specific molecular mechanism of ACE regulating immunity needs to be further explored.

## 5 Conclusion

ACE acupuncture, a traditional Chinese medicine regimen, can protect T lymphocytes, inhibit the degree of damage caused by chemotherapy, and maintain the normal distribution of various subgroups of cells. Therefore, it can potentially improve the immune status and anti-tumor ability of CRC patients, but its specific immune regulatory mechanism still needs further research. In the future, the sample size will be further expanded, and large sample randomized controlled studies will be conducted using a multicenter design to improve the statistical strength and scientific evidence level of the results. Adding more systematic observation indicators, not only focusing on T-lymphocyte changes but also detecting more comprehensive immune function-related indicators, such as immunoglobulins, complement, and inflammatory cytokines, to further comprehensively evaluate the immune regulatory effect. Overall, this study has certain clinical significance in exploring the immune regulatory effects of ACE, but there are still issues such as small sample size, single indicators, and unclear mechanisms that need further optimization to improve the persuasiveness and scientific value of the study.
